# A Review of Resting-State Electroencephalography Analysis in Disorders of Consciousness

**DOI:** 10.3389/fneur.2017.00471

**Published:** 2017-09-11

**Authors:** Yang Bai, Xiaoyu Xia, Xiaoli Li

**Affiliations:** ^1^Institute of Electrical Engineering, Yanshan University, Qinhuangdao, China; ^2^Department of Neurosurgery, PLA Army General Hospital, Beijing, China; ^3^State Key Laboratory of Cognitive Neuroscience and Learning, IDG/McGovern Institute for Brain Research, Beijing Normal University, Beijing, China

**Keywords:** electroencephalography, disorder of consciousness, vegetative state, minimally conscious state, unresponsive wakefulness syndrome

## Abstract

Recently, neuroimaging technologies have been developed as important methods for assessing the brain condition of patients with disorders of consciousness (DOC). Among these technologies, resting-state electroencephalography (EEG) recording and analysis has been widely applied by clinicians due to its relatively low cost and convenience. EEG reflects the electrical activity of the underlying neurons, and it contains information regarding neuronal population oscillations, the information flow pathway, and neural activity networks. Some features derived from EEG signal processing methods have been proposed to describe the electrical features of the brain with DOC. The computation of these features is challenging for clinicians working to comprehend the corresponding physiological meanings and then to put them into clinical applications. This paper reviews studies that analyze spontaneous EEG of DOC, with the purpose of diagnosis, prognosis, and evaluation of brain interventions. It is expected that this review will promote our understanding of the EEG characteristics in DOC.

## Introduction

Following severe damage to the brain, caused by trauma, stroke, or anoxia, patients may fall into a coma (1, 2). When they move out of a coma, they may evolve into a vegetative state (VS) or a minimally conscious state (MCS) according to observable behavioral features (3). Among them, VS (4), or unresponsive wakefulness syndrome (5), is defined by periods of preserved behavioral arousal (6), but unresponsiveness to external stimuli and an absence of awareness (7). MCS shows signs of fluctuating yet reproducible remnants of non-reflex behaviors (8). The disorders of consciousness (DOC) including coma, VS, and MCS pose challenges to clinicians and neuroscientists for diagnosis, treatment, and daily care (3, 9, 10). A correct diagnosis of MCS and VS is of decisive importance for therapeutic strategy making, as patients with MCS generally show greater responses to some treatments (11).

In clinical practice, electroencephalography (EEG) recordings are often used as a tool to help clinicians with diagnoses and prognoses (10, 12). Analyses of resting-state EEG and event-related potential (ERP) are commonly employed (9). An ERP analysis objectively examines sensory and cognitive functions by averaging repeated stimulus-evoked EEG activity (2, 13). Several passive and active paradigms have been used in patients with DOC (13–17). However, the passive paradigms, such as mismatch negativity and somatosensory-evoked potentials, are overly dependent on the basic perceptional function and cortical sensors, which are commonly less preserved in DOC patients following severe brain injuries (2, 18). The active paradigms, such as motor imagery, require the active participation of patients. This poses several problems when working with DOC patients, such as their impaired cognitive function, fluctuations of arousal levels, fatigue, and subclinical seizure activity. EEG recordings taken in a resting state denote spontaneous neural activity, which is relevant to the fundamental brain state (3, 19). Therefore, appropriate features derived from resting-sate EEG may be helpful in monitoring the brain condition of DOC and contribute to decision-making related to these patients’ care. In this paper, we present a review of studies on resting-state EEG in DOC and attempt to improve our knowledge of EEG features in the diagnosis, prognosis, and evaluation of brain interventions in cases of DOC.

## The EEG Analysis for Diagnosis

Since MCS patients are considered to benefit relatively easily from some specific treatments, compared to VS (11, 20), the ability to differentiate an MCS from a VS would offer great value in making decisions about treatment. In clinical practice, many standardized behavioral scales are used in the assessment of consciousness of brain-injured patients, such as the Glasgow Coma Scale (GCS) (21) and the Coma Recovery Scale-Revised (CRS-R) (22). Among them, the GCS is widely used in the early hours of a patient’s admission, and the CRS-R is used throughout the recovery (1). However, a high ratio of misdiagnoses can be caused by clinicians’ subjective judgments, motor function injuries, and patients’ fluctuating levels of awareness (10, 16, 23). Therefore, one of the primary applications of EEG studies in DOC patients is auxiliary diagnosis. Table 1 summarizes the studies we reviewed in this paper.

**Table 1:** Summary of studies using resting-state EEG for diagnosis, prognosis, and evaluation of intervention and basic researches.

Objectives	Literatures	Methods	Subjects	Accuracy/sensitivity/specificity (%)	Main results
Diagnosis	Coleman et al. (24)	Spectrum power ratio	MCS 4, VS 6	–/–/–	VS showed significantly higher EEG power ratio than MCS

	Schnakers et al. (25)	BIS	VS 32, Coma 11	–/75/75	BIS could differentiate unconscious from conscious
	Schnakers et al. (26)		EMCS 13, MCS 30, VS 13, Coma 16	–/–/–	

	Pollonini et al. (27)	Coherence, Granger causality	MCS 7, SND 9	100/–/–	Number of connections within and between brain regions could differentiate MCS from SND

	Sara and Pistoia (28)	ApEn	VS 10, control 10	–/–/–	ApEn was lower in VS than in controls
	Sarà et al. (29)		VS 38, control 40	–/100/97.5	

	Wu et al. (30)	Lempel–Ziv complexity, ApEn, cross-approximate entropy	MCS 16, VS 21, control 30	–/–/–	VS had lowest non-linear indices than MCS and control had highest indices

	Gosseries et al. (31)	State entropy, response entropy	MCS 26, VS 24, Coma 6	–/89/90	EEG entropy of MCS was higher than VS

	Wu et al. (32)	Cross-approximate entropy	MCS 20, VS 30, control 30	–/–/–	Interconnection of local and distant cortical networks in MCS was superior to that of VS

	Landsness et al. (33)	Slow wave activity	MCS 6, VS 5	–/–/–	MCS showed an alternating sleep pattern;VS preserved behavioral sleep but no sleep EEG patterns;

	Leon-Carrion et al. (34)	Coherence, Granger causality	MCS 7, SND 9	–/–/–	MCS showed frontal cortex disconnection from other cortical regions
					Significant difference in full bandwidth coherence between SND and MCS

	Lehembre et al. (35)	Spectrum power, coherence, imaginary part of coherence, phase lag index	MCS 18, VS 10, Acute/subacute 15	–/–/–	VS showed increased delta, decreased alpha power, and lower connectivity than MCS

	King et al. (36)	wSMI	MCS 68, VS 75, CS 24, control 14	–/–/–	wSMI increases as a function separate VS from MCS

	Malinowska et al. (37)	Matching pursuit decomposition, Slow wave activity, K-complexes	LIS 1, MCS 20, VS 11	87/–/–	Sleep EEG patterns correlated with patients’ diagnosis

	Bonfiglio et al. (38)	Blink-related delta oscillations	MCS 5, VS 4, control 12	–/–/–	Patients showed abnormal blink-related delta oscillations

	Lechinger et al. (39)	Spectrum power	MCS 9, VS 8, control 14	–/–/–	Ratios between frequencies (above 8 Hz) and (below 8 Hz) correlated with CRS-R

	Höller et al. (40)	A total of 44 indices	MCS 22, VS 27, control 23	Partial coherence: MCS vs. VS (88), control vs. MCS (96), control vs. VS (98)	Connectivity was crucial for determining the level of consciousness
				Transfer function: MCS vs. VS (80), control vs. MCS (87), control vs. VS (84)	
				Partial coherence: MCS vs. VS (78), control vs. MCS (93), control vs. VS (96)	

	Sitt et al. (18)	Spectrum power, spectral entropy, Kolmogorov–Chaitin complexity, phase locking index, wSMI, permutation entropy	MCS 68, VS 75, CS 24, control 14	Best cross-validated single measure: MCS vs. VS (AUC = 71 ± 4)	
				Whole set of measures: MCS vs. VS (AUC = 78 ± 4)	
				The most discriminative measure was wSMI, which separated VS from MCS	

	Marinazzo et al. (41)	Multivariate Granger causality, transfer entropy	MCS 10, EMCS 5, VS11, control 10	–/–/–	In VS, the central, temporal, and occipital electrodes showed asymmetry between incoming and outgoing information

	Bonfiglio et al. (42)	Blink-related synchronization/desynchronization	MCS 4, VS 5, control 12	–/–/–	Blink-related synchronization/desynchronization could differentiate MCS from VS

	Naro et al. (43)	Spectrum power, LORETA	MCS 7, VS 6, control 10	–/–/–	Alpha was the most significant LORETA data correlating with the consciousness level

	Piarulli et al. (44)	Spectrum power, spectral entropy	MCS 6, VS 6	–/–/–	MCS showed higher theta and alpha, lower delta, higher spectral entropy, and higher time variability than VS

	Thul et al. (45)	Permutation entropy, symbolic transfer entropy	MCS 7, VS 8, control 24	Permutation entropy: Control vs. MCS (Max AUC = 0.74), control vs. VS (Max AUC = 0.91), MCS vs. VS (Max AUC = 0.74)	
				Symbolic transfer entropy: Control vs. MCS (Max AUC = 0.80), control vs. VS (Max AUC = 0.80), MCS vs. VS (Max AUC = 0.71)	

	Chennu et al. (46)	dwPLI, brain network	MCS 66, VS 23, control 26	VS vs. MCS: Alpha participation coefficient (AUC = 0.83, accuracy = 79%), alpha median connectivity (AUC = 0.82), alpha modular span (AUC = 0.78)	
				MCS− vs. MCS+: delta power averaged over all channels (AUC = 0.79)	

Prognosis	Babiloni et al. (47)	Cortical sources estimated by LORETA	VS 50, control 30	Power of alpha source predicted the follow-up recovery	

	Wu et al. (30)	Lempel–Ziv complexity, ApEn, cross-approximate entropy	MCS 16, VS 21, control 30	Non-linear indices of patients who recovered increased than those in non-recovery	

	Fingelkurts et al. (48)	EEG oscillatory microstates	MCS 11, VS 14	Diversity and variability of EEG for non-survivors were significantly lower than for survivors	

	Sarà et al. (29)	ApEn	VS 38, control 40	Patients with lowest ApEn either died or remained in VS, patients with highest ApEn became MCS or partial or full recovery	

	Cologan et al. (49)	Sleep spindles	MCS 10, VS 10	Patients who clinically improved within 6 months have more sleep spindles	

	Arnaldi et al. (50)	Sleep patterns	MCS 6, VS 20	Sleep patterns were valuable predictors of a positive clinical outcome in sub-acute patients	

	Schorr et al. (51)	Spectrum power, coherence	MCS 15, VS 58, control 24	Short- and long-range coherence had a diagnostic value in the prognosis of recovery from VS	

	Wislowska et al. (52)	Spectral power, sleep patterns, permutation entropy	MCS 17, VS 18, control 26	Sleep patterns did not systematically vary between day and night in patients	
				Day–night changes in EEG power spectra and signal complexity were revealed in MCS, but not VS	
				Sleep patterns were linearly related to outcome	

	Chennu et al. (46)	dwPLI, brain network	MCS 66, VS 23, control 26	Delta band connectivity and network had a clear relationship with outcomes	

Treatment evaluation	Williams et al. (53)	Spectrum power, coherence, zolpidem	Patients response in zolpidem 3	Spectral peak of 6–10 Hz with high spatial coherence was a predictor of zolpidem responsiveness	

	Manganotti et al. (54)	Spectrum power, 20 Hz rTMS	MCS 3, VS 3	rTMS over M1 induced long-lasting behavioral and neurophysiological modifications in one MCS patient	

	Carboncini et al. (55)	Spectrum power, phase synchronization, midazolam	MCS 1	Change in the power spectrum was observed after midazolam	
				Midazolam induced significant connectivity changes	

	Cavinato et al. (56)	Coherence, simple sensory stimuli	MCS 11, VS 15	Increase in short-range parietal and long-range fronto-parietal coherences in gamma frequencies was seen in the controls and MCS	
				VS showed no modifications in EEG patterns after stimulation	

	Pisani et al. (57)	Slow wave activity, 5 Hz rTMS	MCS 4, VS 6	Following the real rTMS, a preserved sleep–wake cycle, a standard temporal progression of sleep stages appeared in all MCS but none of VS	

	Naro et al. (58)	Spectrum power, coherence, tACS	MCS 12, VS 14, control 15	TACS entrained theta and gamma oscillations and strengthened the connectivity patterns within frontoparietal networks in all the control, partial MCS, and some VS	

	Naro et al. (59)	Spectrum power, coherence, otDCS	MCS 10, VS 10, control 10	Fronto-parietal networks modulation, theta and gamma power modulation, and coherence increase were paralleled by a transient CRS-R improvement, only in MCS individuals	

	Naro et al. (60)	Lagged-phase synchronization, network parameters, rTMS	MCS 9, VS 11, control 10	Two VS patients showed a residual rTMS-induced modulation of the functional correlations between the default mode network and the external awareness networks, as observed in MCS	

	Bai et al. (61)	Relative power, coherence, biocoherence, SCS with 5, 20, 50, 70, 100 Hz	MCS 11	Significantly altered relative power and synchronization was found in delta and gamma bands after one SCS stimulation using 5, 70, or 100 Hz	
				Bicoherence showed that coupling within delta was significantly decreased after stimulation using 70 Hz	

Basic research	Davey et al. (62)	Spectrum power, coherence	VS 1	Greater low-frequency power, less high-frequency power, and reduced coherence were over the more damaged right hemisphere	

	Babiloni et al. (63)	Spectrum power, LORETA	LIS 13, control 15	Power of delta and alpha was abnormal in LIS	

	King et al. (36)	EEG oscillatory microstates	MCS 7, VS 14	Decreased number of EEG microstate types was associated with altered states of consciousness	
				Unawareness was associated with the lack of diversity in EEG alpha-rhythmic microstates	

	Sitt et al. (18)	Spectrum power	LIS 1, MCS 2, control 5	One MCS and one LIS showed motor imagery task performance through spectral change which was different from control	

	Varotto et al. (64)	Partial directed coherence	VS 18, control 10	VS patients showed a significant and widespread decrease in delta band connectivity, whereas the alpha activity was hyper-connected in the central and posterior cortical regions	

	Chennu et al. (46)	dwPLI, graph theoretic network	MCS 19, VS 13, control 26	Network of patients had reduced local and global efficiency, and fewer hubs in the alpha band	

	Forgacs et al. (65)	Sleep patterns	EMCS 13, MCS 23, VS 8	Patients with evidence of covert command-following had well-organized EEG background and relative preservation of cortical metabolic activity	
	Pavlov et al. (66)		VS 15	Most of VS patients had abnormal sleep patterns	

*ApEn, approximate entropy; wSMI, weighted symbolic mutual information; tACS, transcranial alternating current stimulation; otDCS, oscillatory transcranial direct current stimulation; rTMS, repetitive transcranial magnetic stimulation; SCS, spinal cortical stimulation; CS, conscious patients; SND, severe neurocognitive disorders; LIS, locked-in syndrome; MCS, minimally conscious state; EMCS, emergence from MCS; VS, vegetative state; dwPLI, debiased weighted phase lag index; AUC, area under the receiver operating characteristic curve*.

Spectrum powers have demonstrated the ability to discriminate between MCS and VS. VS patients have shown increased delta power but decreased alpha power, compared to those with MCS (35, 44). In comparison with healthy subjects, VS patients have shown higher delta and theta frequency powers, and both MCS and VS patients have shown decreased alpha power (39). Moreover, the ratios between higher frequencies (alpha + beta) and lower frequencies (delta + theta) have shown a positive correlation with patients’ CRS-R scores (24, 39) and a correlation with regional glucose metabolism in MCS (*n* = 4) (24). Considering the spatial distribution, cortical EEG sources showed that the MCS and VS have significant variations of delta in the frontal region, theta in the frontal and parietal regions, alpha and beta in the central region, and gamma in the parietal region (43).

Spectral entropy analysis has found that the MCS has higher entropy value than the VS (18, 31), and the entropy values were correlated with CRS-R (31). The spectral entropy of the MCS changes over time, and periodicities closely resemble being awake in healthy subjects (44). Therefore, the spectral entropy value and its periodic characteristic have been suggested as potential indices for differentiating the MCS from VS. Some other spectrum-derived indices have been introduced in DOC research, such as BIS. BIS was demonstrated to discriminate between an unconscious state and a conscious one (with a value of 50) in one study (25). It could effectively distinguish the VS from the MCS (26).

Entropy theory has also been applied in the time domain of EEG. Approximate entropy (28–30), Lempel–Ziv complexity (30), permutation entropy (18), and Kolmogorov–Chaitin complexity (18) indices have been proposed to investigate the association of EEG complexity with the consciousness levels of DOC patients. Generally, the VS had lower EEG complexity than the MCS, and the control had the highest (30). Among the indices, Kolmogorov–Chaitin complexity and permutation entropy have been indicated as capable of discriminating the MCS from the VS (18, 45).

Functional connectivity is a crucial method for examining consciousness (40, 67). Among the connectivity methods, coherence was the earliest connectivity measurement used in DOC research (62). The results of one study showed that the frontal regions and their connections with the left temporal and parieto-occipital areas could differentiate the MCS and severe neurocognitive disorders, and this difference was consistent with the results of a Granger causality (27). Similarly, a study of coherence performed by Leon-Carrion et al. showed significant differences in full bandwidth (delta, theta, alpha, and beta) in MCS patients with severe neurocognitive disorders (34). However, the coherence methodology has inherent defects that prevent it from being considered as an ideal method for describing global networks (68, 69). Lehembre et al. compared three connectivity methods (coherence, the imaginary part of coherence, and the phase lag index) and found that significantly lower connectivity of the VS than the MCS could be detected by the imaginary part of coherence and the phase lag index, but failed with coherence (35). Another study addressed 44 indices and proved that partial coherence, directed transfer function, and generalized partial directed coherence were methods with above-chance accuracy for the distinction of an MCS from a VS (with accuracy levels of 0.88, 0.80, and 0.78, respectively) (40).

Furthermore, some other connectivity approaches have been employed, such as weighted symbolic mutual information (wSMI), cross-approximate entropy (32), debiased weighted phase lag index (dwPLI) (46), symbolic transfer entropy, and multivariate Granger causality (41). Among them, wSMI has demonstrated a dissociation with consciousness levels in DOC patients (36), and it was significantly lower in VS in theta and alpha bands (18). Similarly, connectivity and network parameters measured by dwPLI in delta and alpha bands also provided valuable approaches to discriminate different consciousness levels in DOC patients (46).

New approaches using non-strict resting-state EEG might provide new perspectives for finding physiological features that may contribute to diagnoses. Standard EEG patterns in DOC patients showed a difference between the MCS and VS in sleeping states (33). The occurrence of EEG patterns, including sleep spindles, slow wave activity, and the variability of brain rhythms (theta, alpha, and beta), were demonstrated to have significant correlations with the patients’ behavioral diagnoses (37). Bonfiglio et al. proposed that the detection of blink-rated oscillations contributed to the differential diagnoses between the MCS and VS (38, 42). Blink-related delta oscillations linked with awareness of the surrounding environment, which was a criterion for assessing consciousness. The detection of blink-related activity differs from the classical resting-state measurement. However, although it included an event input, the resting-state blinking used in the studies was also a type of spontaneous activity which differed from external stimulus used in ERP.

## The EEG Analysis for Prognosis

The prognosis for survival and recovery of DOC is still difficult under present clinical conditions (70, 71). Generally, the outcomes at 3 and 6 months following the first assessment were selected to observe the predictive performance of the measures. After 3 months of observation, the spectrum power of EEG recordings showed potentially positive performance in predicting the outcomes of a persistent VS (a patient stays in a VS over 1 month after brain injury) (47). Measured by the level of the cognitive functioning scale (LCF), 12 of 50 patients recovered (from LCF I–II to LCF V–VIII). All the patients stayed in a chronic DOC state at the first evaluation. Compared to healthy subjects, the power of alpha in the occipital region showed progressive decay from healthy subjects to recovered patients and then to non-recovered patients. Therefore, the alpha oscillation was implied as a predictor of the possibility of consciousness recovery (47).

Studies using 6 months of observation have shown an association of non-linear analysis indices with the follow-up recovery. Lempel–Ziv complexity, ApEn, and cross-approximate entropy have been suggested as being capable of predicting outcomes of DOC patients (10 of 37 recovered, with Glasgow Outcome Scale scores decreasing to 3). The first evaluation was conducted on patients who stayed in a chronic DOC state after the onset of brain injury for less than 6 months; the patients with increasing indices under painful stimuli had a higher probability of recovery (30). Coincidentally, another study also found that the highest ApEn might correspond to partial or full consciousness improvement at 6 months after the first assessment (29). The prognostic value of resting-state EEG in predicting survival or non-survival 6 months after brain injury was also proven by EEG oscillatory microstate analyses (48). The first EEG recording of the patients was obtained between 14 days and 3 months after acute brain events. The diversity and variability of EEG oscillations and the probability of the appearance of delta, theta (slow and fast), and alpha oscillations were shown to be potential prognostic features in predicting the outcomes of DOC at the group level. In a recent study, 39 of 61 patients had positive outcomes (assessed by Glasgow Outcome Scale-Extended) at 1 year following the first assessment (46). EEG analysis of the patients found that the connectivity and brain network parameters in delta band had a clear relationship with their outcomes. Meanwhile, EEG sleep patterns were also demonstrated valuable predictors of patients’ clinical outcomes (49, 50, 52). Especially, the density of sleep spindles provided significantly predictive and valuable information about the clinical outcomes of DOC patients.

## The EEG Analysis for the Evaluation of Brain Intervention

Due to a variety of etiological, brain injury, and cortical conditions, DOC patients have shown various responses to treatment therapies (20, 72). A precise evaluation of the cerebral responses in the treatment would be helpful for understanding the mechanism of the intervention and facilitate the creation of individual therapeutic strategies. In practice, behavioral changes induced by treatment might be long-lasting accumulated effects that could not be observed immediately. Recently, indices based on EEG analyses were applied to monitor the instantaneous cerebral responses in pharmacological and non-pharmacological brain interventions (16).

Spectrum power, connectivity of coherence, and phase synchronization have been used to assess the cerebral changes of patients in pharmacological treatment (53, 55). For MCS patients who respond to midazolam, spectrum power changes and connectivity changes were found after taking the medication (55). While under zolpidem treatment, all patients showed a distinct low-frequency oscillatory peak at approximately 6–10 Hz over the fronto-central regions (53). Resting-state EEG in non-pharmacological interventions have been investigated in DOC treatment, such as spinal cord stimulation (61), repetitive transcranial magnetic stimulation (rTMS) (54, 57, 60), sensory stimuli (56), transcranial alternating current stimulation (58), and oscillatory transcranial direct current stimulation (59). The fronto-parietal networks of the MCS in the theta and gamma bands have been demonstrated as being responsive to transcranial current stimulation, with little reactivity found in the VS (58, 59). This modulation of a consciousness-related network may suggest more benefits to the MCS than the VS from transcranial current stimulation, and the differential cortical responses between the MCS and VS might provide a stimulus-response approach for diagnoses. Similarly, the different EEG responses between the MCS and VS have also been demonstrated in rTMS, proven by spectrum power (54), complex network parameters (60), and slow wave activity in sleeping (57). In addition, we have attempted to use resting-state EEG as an assistive method for parameter selection in spinal cord stimulations of patients with DOC (61).

## Summary and Conclusion

**Figure 1:**
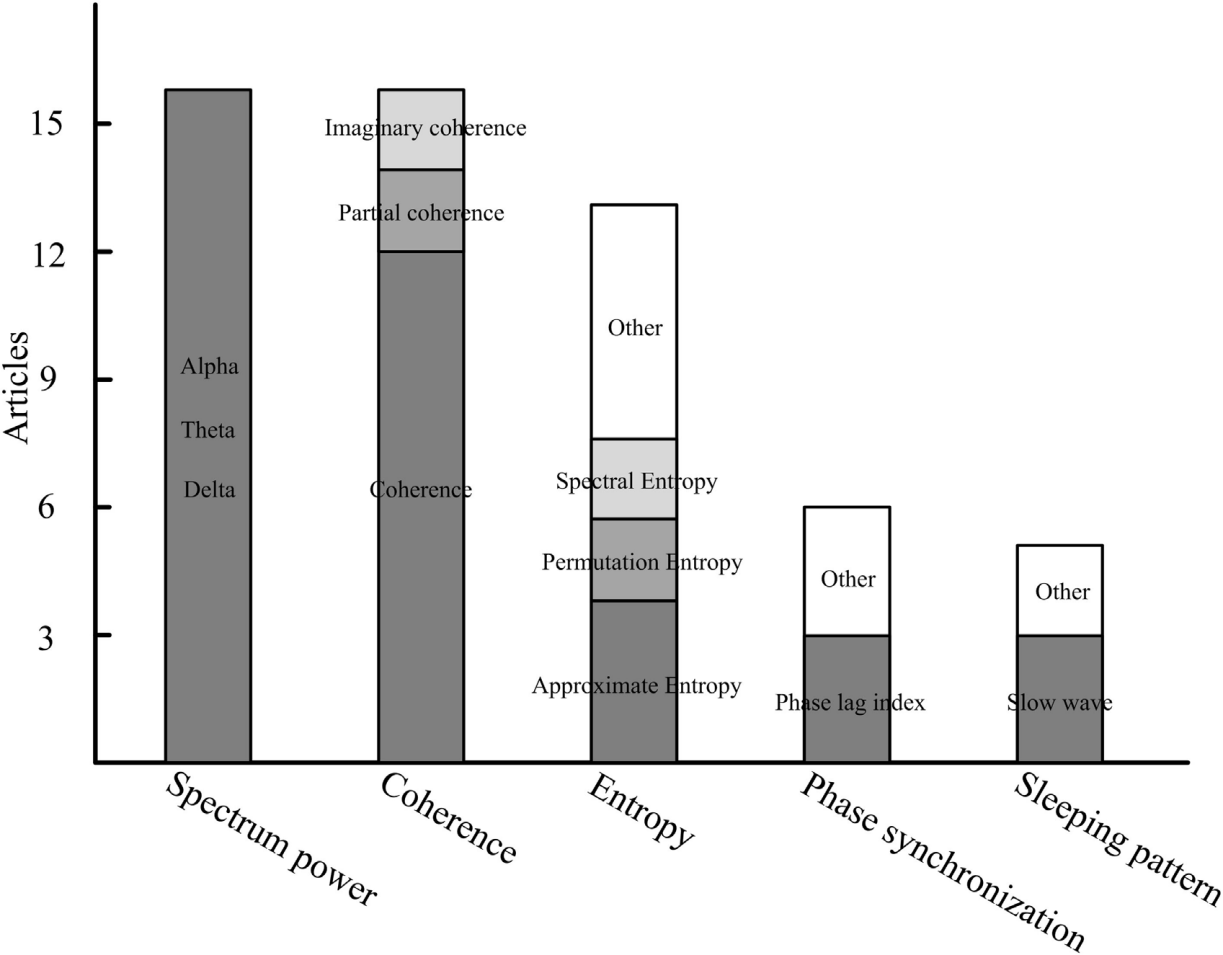
The primary features in resting-state electroencephalography studies of disorders of consciousness.

The characteristics that have been applied in DOC-related studies could be generally classified into five categories: the spectrum, entropy, connectivity, the network, and the sleeping pattern. We summarize the primary features that are frequently used in DOC studies (Figure 1). We found that spectrum power, coherence, and entropy were the most frequently used features in differentiating consciousness levels, predicting follow-up outcome or measuring patients’ cortical response to brain intervention. Comparisons of various methods with multiple indices were performed in two studies (18, 40). Indices derived from spectrum, non-linear analysis, information theory, and functional connectivity were investigated. A discrimination performance of the measures supports power spectrum and functional connectivity as having the best performance in separating the VS from the MCS and healthy subjects (18). In addition, permutation entropy in the theta frequency also has relatively higher classification accuracy in distinguishing the MCS and VS.

Spectral power measures the strength of neuronal oscillations, which depend on the spontaneously activity of underlying oscillators (neurons) (73). Spectral power at some specific frequency can reveal relationships between the activity of groups of neurons and consciousness levels (24, 39). Reviewing the studies, increases of low power (delta and theta), and decreases of high power (alpha) were common spectrum characteristics of patients with DOC. In comparing the MCS and VS, the latter has increased delta and decreased alpha power than the former. Therefore, a power ratio index may be first considered to help us qualitatively assess the consciousness state of patients. In predicting the follow-up outcomes, alpha power should always receive attention. In addition, theta and alpha bands are also critical frequency bands in assessing cortical responses to brain interventions.

Since the neuronal oscillations and synchronization are two essential features of the conscious brain (74), synchronization should be a critical feature in understanding the consciousness of patients with DOC. Synchronization analysis could reveal direct structural connections or indirect information flows, and it could concurrently provide temporal causality and spatial links (2, 75). Non-directed (coherence, the phase locking index, partial directed coherence, the imaginary part of coherence, the dwPLI, cross-approximate entropy, and wSMI) and directed (transfer entropy, symbolic transfer entropy, mutual information, and Granger causality) connectivity measurements were used to reveal the “disconnection” characteristics of patients with DOC (32, 64). Among the measurements, coherence is the most commonly used method. In addition, disconnection between the frontal and other regions, especially the fronto-parietal, was shown to be a significant biomarker, whether assessing the consciousness level or evaluating the brain response to intervention. However, when taking the synchronization feature into actual clinical operation, the reference location, artifact robustness, volume conduction, interesting regions, and cautious physiological explanations should be taken into account.

Similar to EEG complexity in a sleep or anesthesia state (76–78), the complexity measures in DOC were based on the hypothesis that neural activities would be suppressed in a brain of a low consciousness level, and thus fewer components would be included in the EEG signals. EEG complexity, whether measured in the time domain (such as approximate entropy, Lempel–Ziv complexity, Kolmogorov–Chaitin complexity, and permutation entropy) or the frequency domain (BIS and spectral entropy), provided relatively effective and readily comprehensible indices (range of 0–1 or 0–100, with higher value corresponding to higher consciousness level) to describe brain electrical activities under different consciousness states. Therefore, complexity characteristics may have potential value in quantitatively describing the consciousness level of patients with DOC and finally are implanted into monitors for daily caring.

## Author Contributions

YB and XX reviewed the articles and written the manuscript. XL guided the whole work.

## Conflict of Interest Statement

The authors declare that the research was conducted in the absence of any commercial or financial relationships that could be construed as a potential conflict of interest. The reviewer, QN, and handling editor declared their shared affiliation.
